# Utilization due to chronic obstructive pulmonary disease and its predictors: a study using the U.S. National Emergency Department Sample (NEDS)

**DOI:** 10.1186/s12931-015-0319-y

**Published:** 2016-01-06

**Authors:** Jasvinder A. Singh, Shaohua Yu

**Affiliations:** Medicine Service, Birmingham VA Medical Center, Birmingham, AL USA; Department of Medicine at School of Medicine, and Division of Epidemiology at School of Public Health, University of Alabama at Birmingham (UAB), Faculty Office Tower 805B, 510 20th Street S, Birmingham, AL 35294 USA; Department of Orthopedic Surgery, Mayo Clinic College of Medicine, Rochester, MN USA

## Abstract

**Background:**

Previous studies of healthcare utilization for chronic obstructive pulmonary disease (COPD) have focused on time-trends in COPD visits or COPD treatments, or the effect of hospital volume on mortality. Few data are available regarding outcomes after an ED visit (and subsequent hospitalization) for COPD, which are both very common in patients with COPD. Our objective was to assess time-trends and predictors of emergency department and subsequent inpatient health care utilization and charges associated with COPD in the U.S.

**Method:**

We used the 2009-12 U.S. Nationwide Emergency Department Sample (NEDS) to study the incidence of ED visits and subsequent hospitalizations with COPD as the primary diagnosis. We used the 2012 NEDS data to study key patient/hospital factors associated with outcomes, including charges, hospitalization and dischage from hospital to home.

**Results:**

ED visits for COPD as the primary diagnosis increased from 1.02 million in 2009 to 1.04 in 2010 to 1.10 million in 2012 (0.79–0.82 % of all ED visits); respective charges were $2.13, $2.32, and $3.09 billion. In 2012, mean ED charges/visit were $2,812, hospitalization charges/visit were $29,043 and the length of hospital stay was 4.3 days. 49 % were hospitalized after an ED visit. Older age, higher median income, metropolitan residence and comorbidities (diabetes, hypertension, HF, hyperlipidemia, CHD, renal failure and osteoarthritis) were associated with higher risk whereas male sex, Medicaid or self pay insurance status, hospital location in Midwest, South or West U.S. were associated with lower risk of hospitalization. 65.4 % of all patients hospitalized for COPD from ED were discharged home. Older age, comorbidities (diabetes, HF, CHD, renal failure, osteoarthritis) and metropolitan residence were associated with lower odds of discharge to home, whereas male sex, payer other than Medicare, Midwest, South or West U.S. hospital location were associated with higher odds.

**Conclusion:**

Health care utilization and costs in patients with COPD are significant and increasing. COPD constitutes a major public health burden in the U.S. We identified risk factors for hospitalization, costs, and home discharge in patients with COPD that will allow future studies to investigate interventions to potentially reduce COPD-associated utilization.

**Electronic supplementary material:**

The online version of this article (doi:10.1186/s12931-015-0319-y) contains supplementary material, which is available to authorized users.

## Background

Chronic obstructive pulmonary disease (COPD) is associated with significant morbidity and health care costs worldwide [1]. COPD is the third leading cause of mortality in the U.S. and affects >12 million Americans [2, 3]. COPD was associated with $32 billion in costs in 2010 in the U.S. [4]. It frequently leads to inability to work and mobility limitations [5], which poses high societal economic burden [6]. COPD is associated with significant decrements in quality of life [7], worst in those with higher COPD disease severity [8, 9]. In addition to clinical burden, the inability to work due to COPD also poses high economic burden on both patients and governments [6].

Acute exacerbations of COPD are associated with significant morbidity, and decrements in quality of life [10, 11] and lead to frequent ED visits and hospitalizations. Studies have examined factors associated with hospitalization in patients with COPD, mostly in single-center settings or small cohorts [12–14]. One study using a nationally representative U.S. sample found that EDs with higher COPD volume had lower mortality and shorter hospital length of stay [15] compared to those with a lower volume. A previous study using the U.S. National Hospital Ambulatory Medical Care Survey (NHAMCS) from 1993 to 2005 reported 0.6 million COPD visits annually [16], consistent with the estimates reported earlier using the National emergency Department (NEDS) data [15].

Previous studies of COPD ED visits focused on time-trends in visits [17], effect of hospital volume on mortality [15] and trends in treatment [16]. Few, if any data, are available regarding outcomes after an ED visit for COPD. A recent NEDS study comparing COPD with other chronic conditions combined COPD and bronchiectasis and did not provide separate data for COPD [17]. An older study estimated 1.5 million emergency department visits in 2002 in the U.S. [18]. These Centers for Disease Control and Prevention (CDC) data are 15-years old. Thus, contemporary data related to ED and inpatient burden due to COPD in the U.S. are needed, to better understand the current COPD burden on the healthcare system. Our objective was to examine the predictors and estimates of ED and inpatient utilization due to COPD as the primary diagnosis and post-hospitalization disposition, using contemporary NEDS data.

## Methods

### Data source and study population

We used the discharge data from the Nationwide Emergency Department Sample (NEDS), the largest, all-payer U.S. emergency department [ED] database that contains a 20 % stratified sample of ED visits from across the U.S., provided by the Healthcare Cost and Utilization Project (HCUP), Agency for Healthcare Research and Quality [19, 20]. NEDS data is provided by the HCUP State Emergency Department Databases (SEDD) and the State Inpatient Databases (SID) [19, 20] that capture the discharge information on ED visits that do not result vs. that result in an admission to the same hospital, respectively. As an example, 950 U.S. hospitals from 30 states contributed data in 2012.

NEDS is publicly available. NEDS provides appropriate weights to obtain weighted national estimates [20]. For this study, we limited analyses to patients aged 18 and older with an ED visit with COPD as the primary diagnosis. COPD-related visits and hospitalizations were identified using the International Classification of Diseases, ninth revision, Common Modification (ICD-9-CM) code of 491.xx, 492.xx and 496.xx, which have been previously shown to be valid [21]. NEDS contains event-level data but not unique identifiers so that individuals may be represented by multiple visits in any given year. The Institutional Review Board at the University of Alabama at Birmingham (UAB) approved the study.

### Outcomes of interest

We examined the following outcomes of interest: (1) ED discharge disposition (hospitalization vs.routine discharge); (2) ED charges; (3) Inpatient discharge to home (vs. other); (4) Duration of hospital stay; and (5) Total charges (ED and inpatient).

### Covariates

We examined patient and hospital characteristics as covariates. These included age, sex, insurance status, residence [urban vs. rural] and annual median household income estimated using residential zip code. Hospital characteristics included geographical region [Northeast, Midwest, South and West], location in metropolitan or non-metropolitan area, and whether the hospital is teaching vs. non-teaching. For each visit in NEDS, up to 15 ICD-9-CM diagnostic codes, and nine ICD-9-CM procedure codes are provided. We pre-specified certain non-pulmonary comorbidities as potential predictors of outcomes in patients with COPD as the primary reason for ED visit/hospitalization. These comorbidities were chosen due to their common occurrence (coronary heart disease [CHD], heart failure [HF], diabetes, renal failure, hyperlipidemia, hypertension, osteoarthritis [OA], gout).

### Statistical analysis

We calculated summary statistics for key outcomes associated with COPD-related ED-visits and COPD-related hospitalizations across 2009 to 2012. The original 2011 Nationwide Emergency Department Sample (NEDS) included duplicated records in the South and Midwest and a corrected 2011 dataset was not available in amended form for us to analyze. Therefore, most analyses for time-trends used 2009, 2010 and 2012 data. We used the 2012 NEDS data, as the most recent data available, to analyze whether patient and hospital factors were associated with outcomes of COPD-related ED visit, i.e. ED visit with COPD as the primary diagnosis (charges; hospital admission vs. not), COPD-related inpatient admission (length of stay; total charges) and disposition after inpatient admission (discharge to nursing home, total charges).

We examined patient- and hospital characteristics and comorbidities (secondary diagnoses) as potential predictors of these outcomes (see covariate section). We performed multivariable-adjusted logistic regression (discharge disposition, length of hospital stay ≥2 days) or linear regression (charges, duration of hospitalization) using SAS version 9.3 (SAS corporation, Cary, NC, USA). Sensitivity analyses examined the log of hospital stay, hospital stay when dichotomized at 2 days, and the log of total hospital charges, since hospital stay and charges variables were slightly more normal distributed when log-transformed and to examine whether findings were robust or not. Sensitivity models were also performed adding asthma as a pulmonary comorbidity to main models, since it frequently accompanies COPD.

## Results

### Clinical and demographic characteristics

The number of ED visits for COPD increased from 1.02 million in 2009 to 1.04 in 2010 to 1.10 in 2012 (Table 1). There were similar proportions of patients across study years who were female or lived in metropolitan area. Across the study years, the primary payer, hospital region and teaching status were also similar (Table 1).

**Table 1:** Emergency department (ED) visits for COPD as the primary diagnosis in year 2009, 2010 and 2012 NEDS database

	2009 NEDS	2010 NEDS	2012 NEDS
#COPD ED visits (% total)	1,019,276 (0.79)	1,039,825 (0.80)	1,100,378 (0.82)
Age, in years			
Mean (SE)	65.99 (0.15)	65.82 (0.13)	65.49 (0.13)
Median (IQR)	65.88 (55.71, 75.70)	65.68 (55.52, 75.36)	65.04 (55.34, 74.82)
Sex			
Female	561298 (55.13)	573,622 (55.17)	608,895 (55.34)
Patient location (residence)			
Micropolitan/not metro	283,145 (27.95)	280,036 (27.07)	297,501 (27.13)
Metropolitan (large or small)	729,842 (72.05)	754,433 (72.93)	798,895 (72.87)
Median house hold income			
1st quartile (< $38,999)	371,277 (37.46)	373,961 (36.88)	428,687 (39.81)
2nd quartile ($39,000 to $47,999)	307,056 (30.98)	302,423 (29.83)	295,576 (27.45)
3rd quartile ($48,000 to $62999)	198,385 (20.01)	206,248 (20.34)	219,078 (20.34)
4th quartile ($63,000 or more)	114,521 (11.55)	131,230 (12.94)	133,571 (12.40)
Primary payer			
Medicare	643,010 (63.18)	652,143 (62.84)	692,608 (63.02)
Medicaid	137,000 (13.46)	146,288 (14.10)	163,123 (14.84)
Private insurance	142,543 (14.00)	139,120 (13.40)	128,603 (11.70)
Self-pay/no charge	71,418 (7.02)	768,75 (6.92)	84,873 (7.72)
Other	23,818 (2.34)	23,395 (0.49)	29,812 (2.71)
Hospital Region			
Northeast	170,206 (16.70)	175,398 (16.87)	181,739 (16.52)
Midwest	250,323 (24.56)	261,053 (25.11)	268,176 (24.37)
South	457,795 (44.91)	446,524 (42.94)	498,801 (45.33)
West	140,952 (13.83)	156,850 (15.08)	151,661(13.78)
Teaching status of hospital			
Metropolitan non-teaching or non-metro	730,087 (71.63)	721,206 (69.36)	742,700 (67.49)
Metropolitan teaching	289,189 (28.37)	318,619 (30.64)	357,679 (32.51)

*IQR* interquartile range, *SE* standard error

**Fig. 1:**
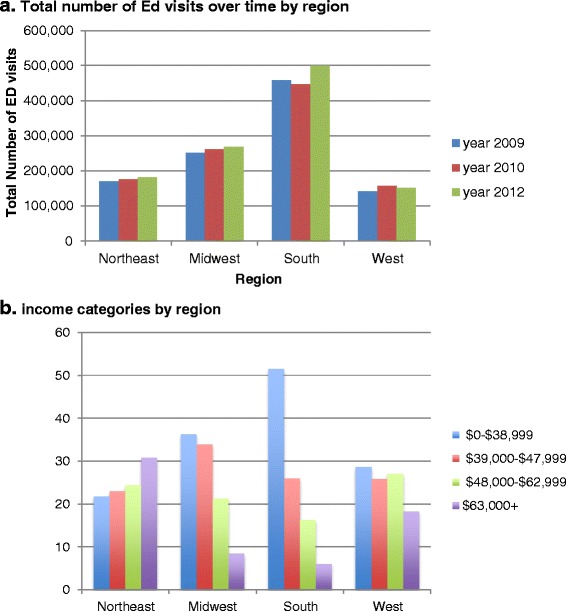
Distribution of the total number of ED visits (**a**) and of income categories (**b**) with COPD as the primary diagnosis by region of residence.* Multivariable model adjusted for gender, race, Charlson score, beta-blockers, diuretics, ACE inhibitors and statins. X-axis represents the respective age, gender and race category and the y-axis hazard ratio. Each column represents the hazard ratio for that patient characteristic. Reference category is no allopurinol use (use = 0 days), marked by hashed line passing through the hazard ratio of 1.00. Error bars represent 95 % confidence interval

The mean age for patients with COPD with ER visit in 2012 was 65.5 years, and 55 % were female (Table 1). 73 % lived in the metropolitan area, Medicare was the primary payer for 63 % and 45 % of all COPD-hospitalizations occurred in hospitals located in the South (Table 1). The number of ED visits over time was stable (Fig. 1a) and income varied significantly by region (Fig. 1b), being lower in Southern US than other regions.

### Utilization and charges for COPD-related visits

COPD ED visits were 0.79–0.82 % of all ED visits (128.9 million, 129.0 million and 134.4 in 2009, 2010 and 2012) during the study period. The ED charges for visits with COPD as the primary diagnosis were $2.13, $2.32, and $3.09 billion in 2009, 2010, and 2012 respectively (Additional file 1: Appendix 1). The total charges for ED and inpatient services in COPD patients who were admitted to the hospital with COPD as the primary diagnosis were $12.5, $12.7, and $14.2 billion in the respective years (Additional file 1: Appendix 1). Total ED and inpatient charges with COPD diagnosis in any position (primary or other) were $121 billion in 2012 (Additional file 1: Appendix 1).

Forty-nine percent were admitted to the hospital from the ED. Of those hospitalized with COPD as the primary diagnosis, 46 % were discharged to home (Additional file 1: Appendix 2). Mean ED charges for ED visits with COPD as primary diagnosis were $2,812 and mean total charges for admitted patients in 2012 were $29,043. Mean length of hospital stay was 4.3 days (Additional file 1: Appendix 2). Outcomes by study year are shown in Additional file 1: Appendix 2.

### Predictors of ED charges and discharge disposition

Older age, male sex, residence in metropolitan area and hospital location in Midwest, South or West U.S. location, were each associated with higher charges for ED visit for COPD in multivariable-adjusted analyses (Additional file 1: Appendix 3).

In univariate analyses, several patient and hospital characteristics and comorbidities were associated with COPD-hospitalization after an ED visit (Additional file 1: Appendix 4). In multivariable-adjusted analyses, older age, higher median household income and metropolitan residence were associated with higher risk whereas male sex, Medicaid or self pay insurance status and hospital location in Midwest, South or Western U.S. were associated with lower risk of hospitalization after an ED visit for COPD (Table 2). Diabetes, hypertension, HF, hyperlipidemia, CHD were associated with 1.2–2.3 fold higher odds, renal failure with 3.1-fold and osteoarthritis with 5.0-fold odds of hospitalization in patients with ED visit with COPD as the primary diagnosis, respectively (Table 2).

**Table 2:** Predictors of hospital admission among patients presenting to ER with COPD as the primary diagnosis using logistic regression

	Univariate		Multivariable-adjusted	
	OR (95 % CI)	*P*-value	OR (95 % CI)	*P*-value
Age				
< 50	Ref		Ref	
50- <65	**2.01 (1.92, 2.09)**	**<0.0001**	**1.45 (1.38, 1.51)**	**<0.0001**
65- <80	**3.42 (3.23, 3.61)**	**<0.0001**	**1.67 (1.57, 1.77)**	**<0.0001**
≥ 80	**4.95 (4.65, 5.27)**	**<0.0001**	**1.90 (1.77, 2.03)**	**<0.0001**
Gender				
Female (ref)	Ref		Ref	
Male	**0.98 (0.96, 1.00)**	**0.0309**	**0.91 (0.89, 0.93)**	**<0.0001**
Median household income				
1st quartile (< $38,999)	Ref		Ref	
2nd quartile ($39,000 to $47,999)	**1.14 (1.06, 1.23)**	**0.0009**	1.08 (1.00, 1.18)	0.0509
3rd quartile ($48,000 to $62999)	**1.40 (1.29, 1.53)**	**<0.0001**	**1.17 (1.07, 1.29)**	**0.0012**
4th quartile ($63,000 or more)	**2.08 (1.86, 2.33)**	**<0.0001**	**1.45 (1.29, 1.63)**	**<0.0001**
Primary payer				
Medicare (ref)	Ref		Ref	
Medicaid	**0.52 (0.49, 0.54)**	**<0.0001**	**0.91 (0.86, 0.96)**	**0.0005**
Private insurance	**0.63 (0.60, 0.66)**	**<0.0001**	0.98 (0.93, 1.03)	0.4990
Self-pay/no charge	**0.30 (0.28, 0.31)**	**<0.0001**	**0.70 (0.66, 0.74)**	**<0.0001**
Other	**0.63 (0.55, 0.72)**	**<0.0001**	1.04 (0.93, 1.15)	0.5203
Patient location (residence)				
Micropolitan/not metropolitan	Ref		Ref	
Metropolitan (large or small)	**1.70 (1.54, 1.89)**	**<0.0001**	**1.42 (1.27, 1.58)**	**<0.0001**
Hospital Region				
Northeast	Ref		Ref	
Midwest	**0.54 (0.45, 0.64)**	**<0.0001**	**0.60 (0.51, 0.71)**	**<0.0001**
South	**0.66 (0.56, 0.79)**	**<0.0001**	**0.80 (0.67, 0.95)**	**0.0106**
West	**0.58 (0.48, 0.69)**	**<0.0001**	**0.63 (0.52, 0.76)**	**<0.0001**
Teaching status of hospital				
Metropolitan non-teaching or non-metropolitan	Ref		Ref	
Metropolitan teaching	**1.34 (1.17, 1.53)**	**<0.0001**	1.14 (0.99, 1.30)	0.0687
Comorbidities				
CHD (ref: no)	**3.77 (3.55, 4.01)**	**<0.0001**	**1.89 (1.80, 1.99)**	**<0.0001**
Hyperlipidemia (ref: no)	**4.30 (3.98, 4.64)**	**<0.0001**	**2.35 (2.19, 2.51)**	**<0.0001**
Renal failure (ref: no)	**6.90 (6.33, 7.53)**	**<0.0001**	**3.10 (2.83, 3.39)**	**<0.0001**
CHF (ref: no)	**3.96 (3.73, 4.21)**	**<0.0001**	**2.13 (2.02, 2.25)**	**<0.0001**
Gout (ref: no)	**4.61 (4.08, 5.22)**	**<0.0001**	**1.87 (1.64, 2.14)**	**<0.0001**
Diabetes (ref: no)	**2.24 (2.14, 2.33)**	**<0.0001**	**1.19 (1.15, 1.24)**	**<0.0001**
Hypertension (ref: no)	**3.50 (3.31, 3.69)**	**<0.0001**	**1.93 (1.84, 2.02)**	**<0.0001**
Osteoarthritis (ref: no)	**6.83 (6.07, 7.67)**	**<0.0001**	**5.02 (4.42, 5.70)**	**<0.0001**

*CHD* coronary heart disease, *CHF* congestive heart failure, *COPD* chronic obstructive pulmonary disease, Significant odds ratios are in bold

### Hospitalization disposition and predictors

In 2012, of all hospitalizations for COPD as the primary diagnosis, 65.4 % were discharged home, 13.4 % to skilled nursing/intermediate facilities, 17.2 % to home health care and 1.1 % died (Additional file 1: Appendix 5). Multivariable-adjusted analyses showed that older age and metropolitan residence were associated with lower odds, whereas male sex, payer other than Medicare, and Midwest, South or West U.S. hospital location were associated with higher odds of discharge to home (Table 3). Comorbidities except hyperlipidemia, hypertension, and gout were associated with lower-odds of discharge to home (Table 3); heart disease and osteoarthritis were not significantly associated. As expected, an opposite direction for associations was noted in multivariable-adjusted analyses for discharge to skilled facilities (Additional file 1: Appendix 6).

**Table 3:** Predictors of discharge to home among patients hospitalized with COPD as the primary diagnosis using logistic regression

	Univariate		Multivariable-adjusted	
	OR (95 % CI)	*P*-value	OR (95 % CI)	*P*-value
Age				
< 50	Ref		Ref	
50- <65	**0.72 (0.67, 0.77)**	**<0.0001**	**0.83 (0.76, 0.90)**	**<0.0001**
65- <80	**0.34 (0.31, 0.37)**	**<0.0001**	**0.51 (0.46, 0.56)**	**<0.0001**
≥ 80	**0.16 (0.14, 0.17)**	**<0.0001**	**0.25 (0.23, 0.28)**	**<0.0001**
Gender				
Female	Ref		Ref	
Male	**1.26 (1.22, 1.30)**	**<0.0001**	**1.22 (1.18, 1.26)**	**<0.0001**
Median house hold income				
1st quartile (< $38,999)	Ref		Ref	
2nd quartile ($39,000 to $47,999)	0.95 (0.89, 1.01)	0.1216	1.03 (0.96, 1.10)	0.3935
3rd quartile ($48,000 to $62999)	**0.81 (0.75, 0.89)**	**<0.0001**	0.97 (0.89, 1.06)	0.4857
4th quartile ($63,000 or more)	**0.65 (0.59, 0.72)**	**<0.0001**	0.99 (0.89, 1.10)	0.8347
Primary payer				
Medicare	Ref		Ref	
Medicaid	**2.36 (2.22, 2.52)**	**<0.0001**	**1.30 (1.22, 1.39)**	**<0.0001**
Private insurance	**2.84 (2.60, 3.10)**	**<0.0001**	**1.89 (1.74, 2.06)**	**<0.0001**
Self-pay/no charge	**6.73 (5.97, 7.59)**	**<0.0001**	**3.01 (2.62, 3.45)**	**<0.0001**
Other	**2.47 (2.13, 2.87)**	**<0.0001**	**1.46 (1.23, 1.74)**	**<0.0001**
Patient location (residence)				
Micropolitan/not metro	Ref		Ref	
Metropolitan (large or small)	**0.79 (0.73, 0.86)**	**<0.0001**	**0.88 (0.81, 0.96)**	**0.0049**
Hospital Region				
Northeast	Ref		Ref	
Midwest	**1.55 (1.36, 1.76)**	**<0.0001**	**1.29 (1.11, 1.49)**	**0.0007**
South	**1.76 (1.57, 1.97)**	**<0.0001**	**1.52 (1.33, 1.73)**	**<0.0001**
West	**1.83 (1.59, 2.11)**	**<0.0001**	**1.65 (1.43, 1.90)**	**<0.0001**
Teaching status of hospital				
Metropolitan non-teaching or non-metropolitan	Ref		Ref	
Metropolitan teaching	0.92 (0.83, 1.02)	0.1040	0.99 (0.90, 1.10)	0.8999
Comorbidities				
CHD (ref: no)	**0.79 (0.77, 0.82)**	**<0.0001**	1.01 (0.98, 1.05)	0.5676
Hyperlipidemia (ref: no)	**1.06 (1.03, 1.10)**	**0.0008**	**1.22 (1.18, 1.27)**	**<0.0001**
Renal failure (ref: no)	**0.60 (0.57, 0.62)**	**<0.0001**	**0.88 (0.85, 0.92)**	**<0.0001**
CHF (ref: no)	**0.50 (0.48, 0.51)**	**<0.0001**	**0.67 (0.65, 0.69)**	**<0.0001**
Gout (ref: no)	**0.87 (0.80, 0.94)**	**0.0007**	**1.14 (1.04, 1.24)**	**0.0036**
Diabetes (ref: no)	**0.88 (0.85, 0.91)**	**<0.0001**	**0.91 (0.88, 0.94)**	**<0.0001**
Hypertension (ref: no)	**0.88 (0.85, 0.90)**	**<0.0001**	**1.06 (1.03, 1.09)**	**0.0001**
Osteoarthritis (ref: no)	**0.84 (0.80, 0.89)**	**<0.0001**	0.96 (0.92, 1.01)	0.1430
Length of stay, in days	**0.83 (0.83, 0.84)**	**<0.0001**	**0.85 (0.84, 0.85)**	**<0.0001**

*CHD* coronary heart disease, *CHF* congestive heart failure, *COPD* chronic obstructive pulmonary disease, Significant odds ratios are in bold

### Length of hospital stay and charges

Multivariable-adjusted linear regression showed that older age and metropolitan location were associated with longer stay in patients hospitalized with COPD as the primary diagnosis whereas male sex, non-Medicare primary payer, hospital location other than Northeast were associated with shorter hospital stay (Table 4). Most comorbidities except hyperlipidemia, gout and hypertension were associated with longer hospital stay (Table 4); heart disease, diabetes and osteoarthritis were not significantly associated.

**Table 4:** Predictors of duration of hospital stay among patients with COPD who were admitted to the hospital after presenting to ED with COPD as the primary diagnosis using linear regression

	Univariate		Multivariable-adjusted	
	B-estimate (95 % CI)	*P*-value	B-estimate (95 % CI)	*P*-value
Age				
< 50	Ref		Ref	
50- <65	**0.52 (0.43,0.61)**	**<0.0001**	**0.50 (0.40, 0.59)**	**<0.0001**
65- <80	**1.00 (0.89,1.10)**	**<0.0001**	**0.82 (0.70, 0.93)**	**<0.0001**
≥ 80	**1.18 (1.06,1.31)**	**<0.0001**	**0.85 (0.73, 0.98)**	**<0.0001**
Gender				
Female	Ref		Ref	
Male	**-0.28 (-0.34, -0.23)**	**<0.0001**	**-0.29 (-0.34, -0.24)**	**<0.0001**
Median house hold income				
1st quartile (< $38,999)	Ref		Ref	
2nd quartile ($39,000 to $47,999)	0.00 (-0.10, 0.10)	0.9732	-0.03 (-0.12, 0.07)	0.5973
3rd quartile ($48,000 to $62999)	0.05 (-0.06, 0.15)	0.3623	-0.07 (-0.17, 0.03)	0.1951
4th quartile ($63,000 or more)	**0.40 (0.20, 0.59)**	**0.0001**	0.11 (-0.05, 0.28)	0.1895
Primary payer				
Medicare (ref)	Ref		Ref	
Medicaid	**-0.42 (-0.52, -0.32)**	**<0.0001**	-0.06 (-0.16, 0.04)	0.2685
Private insurance	**-0.52 (-0.61, -0.43)**	**<0.0001**	**-0.26 (-0.34, -0.17)**	**<0.0001**
Self-pay/no charge	**-1.19 (-1.29, -1.09)**	**<0.0001**	**-0.74 (-0.85, -0.64)**	**<0.0001**
Other	**-0.61 (-0.80, -0.42)**	**<0.0001**	**-0.26 (-0.42, -0.10)**	**0.0013**
Patient location (residence)				
Micropolitan/not metro	Ref		Ref	
Metro (large or small)	**0.35 (0.23, 0.47)**	**<0.0001**	**0.28 (0.15, 0.41)**	**<0.0001**
Hospital Region				
Northeast	Ref		Ref	
Midwest	**-0.79 (-1.02, -0.57)**	**<0.0001**	**-0.67 (-0.89, -0.45)**	**<0.0001**
South	**-0.43 (-0.65, -0.21)**	**0.0001**	**-0.27 (-0.48, -0.07)**	**0.0103**
West	**-0.80 (-1.03, -0.58)**	**<0.0001**	**-0.76 (-0.98, -0.54)**	**<0.0001**
Teaching status of hospital				
Metropolitan non-teaching or non-metropolitan	Ref		Ref	
Metropolitan teaching	**0.15 (0.01, 0.30)**	**0.0363**	0.03 (-0.12, 0.18)	0.7276
Comorbidities				
CHD (ref: no)	**0.16 (0.10, 0.21)**	**<0.0001**	-0.03 (-0.09, 0.02)	0.2051
Hyperlipidemia (ref: no)	**-0.20 (-0.25, -0.14)**	**<0.0001**	**-0.30 (-0.35, -0.25)**	**<0.0001**
Renal failure (ref: no)	**0.51 (0.43, 0.58)**	**<0.0001**	**0.26 (0.19, 0.33)**	**<0.0001**
CHF (ref: no)	**0.84 (0.78, 0.90)**	**<0.0001**	**0.73 (0.66, 0.79)**	**<0.0001**
Gout (ref: no)	-0.02 (-0.17, 0.13)	0.8001	**-0.24 (-0.39, -0.08)**	**0.0029**
Diabetes (ref: no)	**0.12 (0.06, 0.17)**	**<0.0001**	0.03 (-0.02, 0.08)	0.2479
Hypertension (ref: no)	-0.00 (-0.06, 0.05)	0.9132	**-0.15 (-0.21, -0.09)**	**<0.0001**
Osteoarthritis (ref: no)	-0.01 (-0.10,0.09)	0.8771	-0.07 (-0.16, 0.03)	0.1554

*CHD* coronary heart disease, *CHF* congestive heart failure, *COPD* chronic obstructive pulmonary disease, Significant beta coefficients are in bold. A negative coefficient indicates a shorter hospital stay and positive coefficient, a longer hospital stay

In multivariable-adjusted analyses, older age, higher income, metropolitan location, Western U.S. hospital location, and the presence of renal failure, HF and diabetes were associated higher total (ED + inpatient) hospital charges (Table 5). Male sex, non-Medicare primary payer, and a few comorbidities were associated with lower hospital charges (Table 5).

**Table 5:** Predictors of total (ED and inpatient) hospital charges in patients with COPD who were admitted to the hospital after presenting to ED with COPD as the primary diagnosis using linear regression

	Univariate		Multivariable-adjusted	
	B-estimate (95 % CI)	*P*-value	B-estimate (95 % CI)	*P*-value
Age				
< 50	Ref		Ref	
50- <65	**3891.1 (2985.4, 4796.7)**	**<0.0001**	**3117.53 (2219.7, 4015.4)**	**<0.0001**
65- <80	**6652.1 (5449.1, 7855.1)**	**<0.0001**	**4176.16 (2912.7, 5439.7)**	**<0.0001**
≥ 80	**7109.4 (5582.7, 8636.1)**	**<0.0001**	**2974.40 (1606.2, 4342.6)**	**<0.0001**
Gender				
Female	Ref		Ref	
Male	-426.92 (-1051.0, 197.2)	0.1797	**-665.99 (-1277.8, -54.2)**	**<0.0001**
Median household income				
1st quartile (< $38,999)	Ref		Ref	
2nd quartile ($39,000 to $47,999)	371.8 (-1261.3, 2005.0)	0.6550	-273.8 (-1882.1, 1334.5)	0.7383
3rd quartile ($48,000 to $62999)	**4658.7 (2156.6, 7160.9)**	**0.0003**	**2250.3 (34.0, 4466.6)**	**0.0466**
4th quartile ($63,000 or more)	**7493.74 (3997.5, 10990.0)**	**<0.0001**	**4614.2 (1284.2, 7944.2)**	**0.0067**
Primary payer				
Medicare (ref)	Ref		Ref	
Medicaid	**-2014.3 (-3242.1, -786.4)**	**0.0013**	-963.2 (-2007.8, 81.3)	0.0707
Private insurance	**-3725.8 (-4972.1, -2479.4)**	**<0.0001**	**-2264.3 (-3574.4, -954.1)**	**0.0007**
Self-pay/no charge	**-7134.2 (-8265.9, -6002.6)**	**<0.0001**	**-4291.1 (-5496.3, -3085.8)**	**<0.0001**
Other	-2313.6 (-4674.1, 47.0)	0.0547	**-2427.7 (-4355.3, -500.1)**	**0.0136**
Patient location (residence)				
Micropolitan/not metro	Ref		Ref	
Metro (large or small)	**9681.5 (7392.3, 11970.6)**	**<0.0001**	**8000.2 (5631.0, 10369.3)**	**<0.0001**
Hospital Region				
Northeast	Ref		Ref	
Midwest	-5464.4 (-9760.8, 1168.1)	0.0127	-3131.5 (-7348.2, 1085.1)	0.1453
South	257.9 (-4333.9, 4849.7)	0.9122	3009.9 (-1643.7, 7663.6)	0.2046
West	**17022.9 (11248.1, 22797.8)**	**<0.0001**	**18103.3 (12310.9, 23895.8)**	**<0.0001**
Teaching status of hospital				
Metropolitan non-teaching or non-metropolitan	Ref		Ref	
Metropolitan teaching	181.7 (-3059.2, 3422.7)	0.9124	-461.9 (-3862.1, 2938.4)	0.7898
Comorbidities				
CHD (ref: no)	**2094.6 (1516.9, 2672.4)**	**<0.0001**	**892.91 (347.7, 1438.1)**	**0.0014**
Hyperlipidemia (ref: no)	**-1748.2 (-2335.2, -1161.2)**	**<0.0001**	**-2162.7 (-2709.3, -1616.1)**	**<0.0001**
Renal failure (ref: no)	**4756.1 (3906.7, 5605.5)**	**<0.0001**	**2414.5 (1672.6, 3156.4)**	**<0.0001**
CHF (ref: no)	**7385.2 (6410.1, 8360.3)**	**<0.0001**	**6489.5 (5491.6, 7487.4)**	**<0.0001**
Gout (ref: no)	-1128.9 (-2562.4, 304.6)	0.1225	**-3127.6 (-4587.4, -1667.8)**	**<0.0001**
Diabetes (ref: no)	**1474.4 (853.5, 2095.3)**	**<0.0001**	**844.6 (247.4, 1441.7)**	**0.0056**
Hypertension (ref: no)	329.1 (-310.1, 968.2)	0.3125	**-674.9 (-1341.2, -8.53)**	**0.0471**
Osteoarthritis (ref: no)	**-2524.4 (-3469.5, -1579.3)**	**<0.0001**	**-2153.9 (-3023.9, -1283.9)**	**<0.0001**

*CHD* coronary heart disease, *CHF* congestive heart failure, *COPD* chronic obstructive pulmonary disease, Significant beta estimates are in bold

Sensitivity analyses examining the log of hospital stay (Additional file 1: Appendix 7), hospital stay when dichotomized at 2 days (Additional file 1: Appendix 8), or log of total hospital charges (Additional file 1: Appendix 9) showed similar results as above.

### Impact of co-existing asthma on outcomes

When the main multivariable-adjusted models were additionally adjusted for the presence of asthma, we found that the odds of hospital admission from ED were not increased, 0.91 (0.79, 1.05; *p* = 0.19), but odds of discharge to home were higher, 1.43 (1.29, 1.59); *p* < 0.0001). Asthma was significantly associated with longer duration of hospital stay in those hospitalized, but hospital charges were not significantly different: beta coefficients 0.29 (0.15, 0.43; *p* < 0.0001) and 947.4 (-658.9, 2553.6; *p* = 0.24).

## Discussion

In this study using the NEDS, a U.S. representative national sample, we studied utilization of ED and inpatient resources with COPD as the primary diagnosis. We found that COPD was associated with >1 million ED visits and over half a million hospitalizations in 2012. Our estimates for the overall number of ED visits and inpatient hospitalizations related to COPD are similar to those reported in a study that combined COPD and bronchiectasis [17]. A previous study of trends in COPD treatment in the ED showed higher concordance with treatment recommendations in the recent years [16], indicating that quality of COPD care in the ED may be improving over time. We also examined factors associated with health resource utilization and charges for ED and inpatient visits due to COPD. Several findings merit further discussion.

First, we found that several sociodemographic, comorbidity, and hospital factors were associated with the risk of hospitalization among COPD patients who presented to ED with COPD as the primary diagnosis. Previous studies have focused on pulmonary function status and COPD medications as predictors of COPD hospitalization and associated costs [22, 23], but did not examine socio-demographic and hospital characteristics in detail. Our study examined these key characteristics and made important observations.

We found that older age, median household income above $48,000, metropolitan residence and the presence of comorbidities were each associated with higher risk of hospitalization. The association of older age with higher risk of COPD hospitalization is not surprising and confirms earlier similar findings from studies of smaller sample sizes [12, 24], now in a larger, representative U.S. sample. The association of higher median household income with a higher risk of COPD hospitalization/costs in interesting and extends a similar finding from other chronic conditions [25, 26] to COPD. Since models were adjusted for insurance type, the income effect is independent of insurance status and type. It is possible that income reflects education level or health literacy level, both of which might impact the risk of hospitalization. The association of female sex with higher odds of COPD hospitalization is consistent with a similar finding from Danish population-based study of 1.5-fold higher risk [27]. Medicaid or self-pay insurance status, hospital location in Midwest, South or Western U.S. were associated with lower risk of hospitalization after ED visit for COPD. The regional differences may be related to region-specific practices, differing severity of COPD and/or coexistent comorbidities by region, distance to nearest medical center etc. To our knowledge, our study is the one of the first U.S. studies to comprehensively examine the association of important socio-demographic, comorbidity and hospital characteristics and factors with COPD-hospitalization.

Comorbidities were risk factors for COPD hospitalizations. Diabetes, hypertension, heart failure, hyperlipidemia, and CHD were each associated with 1.2–2.3 fold higher odds of COPD hospitalization. Previous literature examining the association of comorbidities with COPD admission is somewhat contradictory. Coronary artery disease and diabetes mellitus were significant risk factors for COPD admission in some studies [28, 29], but other studies found no association of comorbidities with COPD readmission [30–33]. Potential reasons for differences in findings of previous studies are differences in comorbidities adjusted in the analyses, study settings, and omission of important confounders in some studies. General interventions such as physical rehabilitation can improve outcomes of patients hospitalized with COPD [34]. Other specific interventions and management strategies targeting these comorbidities (diabetes, hypertension, HF, CHD etc.) in patients with COPD might reduce COPD-associated inpatient utilization.

We found that renal failure was associated with 3.1-times higher odds and osteoarthritis with 5.0-fold higher odds of hospitalization among COPD patients who presented to ED. To our knowledge, these findings are novel. Renal failure has been previously associated with higher mortality in hospitalized COPD patients, but risk of hospitalization was not assessed [35–37]. Whether the increased hospitalization risk is due to the presence of renal failure or its worsening during the treatment of COPD exacerbation with corticosteroids and antibiotics in the ED, cannot be determined from our study. Similar to our observation of $4,756 unadjusted higher total charges in COPD patients with renal failure, a recent study by Manino et al. reported that the average all-cause total health-care costs from the index date to 360 days after the index date were highest for patients with chronic kidney disease, by $41,288 [38]. Therefore, future studies need to examine whether optimization of renal function by adequate hydration, medication management and optimization of renal function during a COPD flare and its treatment may help in reducing this risk of COPD hospitalization.

Osteoarthritis is associated with significant disability [39], loss of independence and higher health care utilization [40] in general. Difficulty in ambulation and daily activities due to concomitant osteoarthritis and associated frailty may contribute to a higher risk of hospitalization in COPD patients who also have difficulty in breathing. Use of assistive devices, exercises and self-management strategies reduce disability in osteoarthritis [41–43]; newer methods such as neuromuscular electrical stimulation may be helpful in some patients [44, 45]. Future studies should examine if early diagnosis and optimal treatment of osteoarthritis in patients with COPD, focusing on inexpensive and commonly available non-surgical interventions for osteoarthritis, can reduce the risk of COPD hospitalization.

As noted above, our study findings of the association of various socio-demographic, comorbidity and hospital characteristics with COPD-hospitalization add to the current knowledge. Factors such as long term oxygen therapy, low health status, poor health related quality of life and inadequate physical activity [22] and body mass index, airflow obstruction, dyspnea, and exercise capacity (BODE) index [46] are associated with COPD hospitalization, and can predict hospital length of stay in and mortality in patients with COPD [47–50]. Our study identified additional risk factors for COPD hospitalization. This new knowledge can help clinicians risk stratify COPD patients and target those with poorest prognosis.

Another finding was that older age, diabetes, HF and renal failure were associated with lower odds and male sex with higher odds of discharge to home (vs. nursing home/facilities) in hospitalized COPD patients. These findings are not unexpected; however, we are unaware of any studies with a representative U.S. sample that have explored this aspect of COPD care. Thus, these findings are novel. It remains to be seen whether optimization of HF, renal failure, and diabetes in COPD patients who are admitted to the hospital can increase the proportion discharged to home vs. skilled facilities.

Study findings must be interpreted considering study limitations. These findings are likely only applicable to ED-associated hospitalizations, not all hospitalizations. Due to the nature of NEDS database that captures data at the encounter level, rather than patient level, we are unable to examine risk factors for COPD readmissions, which are of interest clinically and for healthcare policy. We believe that at least some COPD admissions are readmissions. NEDS database does not link the ED visit to inpatient admission to another hospital, and we would likely miss this relatively uncommon event, since linkages of ED to hospitalization are at a hospital level. This underestimation is possible, but it is likely to be an uncommon or rare event. NEDS does not provide disease severity variables to adjust in the analyses, which can lead to residual confounding. Generalizability of findings to other countries may not be possible, since health care settings differ. Detailed assessments of COPD medications and their associations with COPD-related utilization would have provided additional insights; however, these data were not available in NEDs and this was beyond the scope of this study.

Our study has several strengths. NEDS is the largest, publically available U.S. sample of ED visits, therefore the findings are representative of and applicable to the U.S. population. We adjusted for several covariates and confounders including patient and hospital characteristics in patients with COPD to obtain unbiased estimates.

## Conclusions

In conclusion, in this study of 2009-2012 NEDS data of patients presenting to the ED with COPD as the primary diagnosis, we studied patient discharge disposition from the ED and ED costs and for those admitted to the hospital, length of hospital stay, discharge disposition and costs. We found that older age, metropolitan residence and comorbidities (diabetes, HF, CHD, renal failure and osteoarthritis) were associated with higher risk of hospitalization and lower odds of discharge to home. Higher income was also associated with higher risk of hospitalization. We also identified other patient characteristics associated with hospital charges. Studies in the future should examine whether interventions targeting these modifiable factors can improve COPD outcomes.

### IRB approval

The University of Alabama at Birmingham’s Institutional Review Board approved this study and all investigations were conducted in conformity with ethical principles of research.

## Additional file

Additional file 1:
**Appendix 1.** Descriptive for the main ED visit-related outcomes with COPD diagnosis. **Appendix 2.** Patient Outcomes for those presenting to ED with COPD as the primary diagnosis. **Appendix 3.** Predictors of ED hospital charges among patients presenting to ER with COPD as the primary diagnosis using linear regression. **Appendix 4.** Characteristics of patients with COPD ED visits with and without hospitalization. **Appendix 5.** Outcomes of patients after hospital admission with COPD as primary or primary/secondary diagnosis. **Appendix 6.** Predictors of Discharge to nursing home/skilled nursing facility among patients who were admitted to the hospital with COPD as the primary diagnosis after presenting to ED, using logistic regression. **Appendix 7.** Predictors of Log of duration of hospital stay among patients with COPD who were admitted to the hospital with COPD as the primary diagnosis after presenting to ED, using linear regression. **Appendix 8.** Predictors of Duration of stay (length of stay >2; reference, length of stay ≤2) among patients who were admitted to the hospital with COPD as the primary diagnosis after presenting to ED, using logistic regression. **Appendix 9.** Predictors of log of Total hospital charges (ED plus inpatient) among patients with COPD who were admitted to the hospital using linear regression. (DOCX 58 kb)
