# Supplementary material for: Utilization due to chronic obstructive pulmonary disease and its predictors: a study using the U.S. National Emergency Department Sample (NEDS)
Source: Respir Res. 2016 Jan 6;17:1. doi: 10.1186/s12931-015-0319-y (PMC4702346; doi:10.1186/s12931-015-0319-y)
Supplement: Additional file 1: — Appendix 1. Descriptive for the main ED visit-related outcomes with COPD diagnosis. Appendix 2. Patient Outcomes for those presenting to ED with COPD as the primary diagnosis. Appendix 3. Predictors of ED hospital charges among patients presenting to ER with COPD as the primary diagnosis using linear regression. Appendix 4. Characteristics of patients with COPD ED visits with and without hospitalization. Appendix 5. Outcomes of patients after hospital admission with COPD as primary or primary/secondary diagnosis. Appendix 6. Predictors of Discharge to nursing home/skilled nursing facility among patients who were admitted to the hospital with COPD as the primary diagnosis after presenting to ED, using logistic regression. Appendix 7. Predictors of Log of duration of hospital stay among patients with COPD who were admitted to the hospital with COPD as the primary diagnosis after presenting to ED, using linear regression. Appendix 8. Predictors of Duration of stay (length of stay >2; reference, length of stay ≤2) among patients who were admitted to the hospital with COPD as the primary diagnosis after presenting to ED, using logistic regression. Appendix 9. Predictors of log of Total hospital charges (ED plus inpatient) among patients with COPD who were admitted to the hospital using linear regression. (DOCX 58 kb) [file 12931_2015_319_MOESM1_ESM.docx]

Additional file 1

Appendix 1. Descriptive for the main ED visit-related outcomes with COPD diagnosis

|  | **2009 NEDS** | **2010 NEDS** | **2012 NEDS** |
| --- | --- | --- | --- |
| All COPD ED visits with COPD as primary Diagnosis, weighted n(%) of total ED visits | **1,019,276 (0.79)** | **1,039,825 (0.80)** | **1,100,378 (0.82)** |
| Total ED charges ($) | 2,127,534,795 | 2,319,859,973 | 3,095,308,295 |
|  |  |  |  |
| All COPD ED visits (not admitted to the same hospital) with COPD as primary Dx | 462,679 | 489,222 | 559,997 |
| Total ED charges ($) | 1,319,865,876 | 1,434,609,269 | 2,041,065,866 |
|  |  |  |  |
| All COPD inpatient admissions with COPD as primary diagnosis for those with ED visits |  |  |  |
| Total inpatient charges ($) | 12,540,130,410 | 12,741,504,023 | 14,159,089,164 |
| Duration of hospital stay, in days  Mean (SE) | 4.55 (0.03) | 4.40 (0.03) | 4.28 (0.03) |
| Total charges for ED and inpatient services ($) | 13,867,614,255 | 14,181,330,868 | 15,694,314,426 |
|  |  |  |  |
| All COPD inpatient admissions with COPD in any position for those with ED visits |  |  |  |
| Total inpatient charges ($) | 96,768,404,330 | 100,583,917,728 | 109,125,053,268 |
| Duration of hospital stay, in days mean (SE) | 5.58 (0.04) | 5.38 (0.04) | 5.22 (0.04) |
| Total charges for ED and inpatient services ($) | 106,091,665,056 | 110,831,785,980 | 121,332,899,424 |

Appendix 2. Patient Outcomes for those presenting to ED with COPD as the primary diagnosis

|  | **2009 NEDS** | **2010 NEDS** | **2012 NEDS** |
| --- | --- | --- | --- |
| ED disposition* |  |  |  |
| Discharged (routine) | 421,055 (41.31) | 440,819 (42.39) | 507,654 (46.13) |
| Admitted | 556,597(54.61) | 550,603 (52.95) | 540,382 (49.11) |
| Transferred | 29,302 (2.87) | 34,709 (3.33) | 36,966 (3.36) |
| Home health care | 2,591 (0.25) | 1,645 (0.16) | 3,141 (0.29) |
| Against medical advice | 8,326 (0.82) | 8,865 (0.85) | 10,747 (0.98) |
| Unknown | 928 (0.09) | 2,679 (0.26) | 1,092 (1.00) |
| ED charges per ED visit, in US$, |  |  |  |
| Mean (SE) | 2087.3 (40.34) | 2231.01 (43.60) | 2812.95 (77.72) |
| Median (IQR) | 1,449.96  (875.52, 2353.84) | 1,631.75  (1022.76, 2611.48) | 1,948.85  (1214.15, 3207.00) |
| Length of hospital stay for admitted patients, in days |  |  |  |
| Mean (SE) | 4.55 (0.03) | 4.40 (0.03) | 4.28 (0.03) |
| Median (IQR) | 3.12 (1.81, 5.08) | 2.98 (1.72, 4.90) | 2.92 (1.66, 4.80) |
| Total charges per visit for admitted patients, US$) |  |  |  |
| Mean (SE) | 24,915 (530.81) | 25,756 (541.67) | 29,043 (750.61) |
| Median (IQR) | 16,837  (10311, 28554) | 17,841  (10970, 29903) | 19,899  (12055, 34053) |
| IQR, Interquartile range; SE, standard error | | | |

Appendix 3. Predictors of ED hospital charges among patients presenting to ER with COPD as the primary diagnosis using linear regression

|  | Univariate |  | Multivariable-adjusted |  |
| --- | --- | --- | --- | --- |
|  | B-estimate* (95% CI) | P-value | B-estimate* (95% CI) | P-value |
| Age |  |  |  |  |
| <50 | Ref |  | Ref |  |
| 50- <65 | **195.44 (102.31, 288.56)** | **<0.0001** | **197.53 (118.14,276.92)** | **<0.0001** |
| 65- <80 | **132.93 (14.33, 251.53)** | **0.0281** | **172.18 (86.54,257.81)** | **<0.0001** |
| ≥80 | 2.86 (-155.64, 161.37) | 0.9717 | **120.21 (18.35,222.06)** | **0.0208** |
| Gender |  |  |  |  |
| Female (ref) | Ref |  | Ref |  |
| Male | **44.82 (7.35, 82.30)** | **0.0191** | **46.77 (9.95,83.59)** | **0.0128** |
| Median house hold income |  |  |  |  |
| 1st quartile (< $38,999) | Ref |  | Ref |  |
| 2nd quartile ($39,000 - $47,999) | -3.33 (-160.02, 153.36) | 0.9667 | -33.31 (-185.38,118.75) | 0.6673 |
| 3rd quartile ($48,000 - $62999) | 358.01 (-95.51, 811.52) | 0.1216 | 279.02 (-87.11,645.14) | 0.1351 |
| 4th quartile ($63,000 or more) | -170.86 (-442.93, 101.20) | 0.2180 | -113.91 (-443.40,215.57) | 0.4975 |
| Primary payer |  |  |  |  |
| Medicare (ref) | Ref |  | Ref |  |
| Medicaid | 6.74 (-135.42, 148.90) | 0.9259 | -16.94 (-133.18,99.29) | 0.7749 |
| Private insurance | 68.94 (-21.30, 159.19) | 0.1341 | 17.45 (-83.51,118.41) | 0.7345 |
| Self-pay/No charge | **105.11 (1.44, 208.78)** | **0.0469** | -1.41 (-100.39,97.57) | 0.9777 |
| Other | 38.59 (-238.99, 316.17) | 0.7850 | -35.28 (-292.50,221.93) | 0.7878 |
| Patient location (residence) |  |  |  |  |
| Micropolitan/not metro | Ref |  | Ref |  |
| Metro (large or small) | **424.83 (181.70, 667.96)** | **0.0006** | **510.82 (247.19,774.45)** | **0.0002** |
| Hospital Region |  |  |  |  |
| Northeast | Ref |  | Ref |  |
| Midwest | **593.83 (62.33, 1125.32)** | **0.0286** | **637.69 (84.20,1191.19)** | **0.0240** |
| South | **616.38 (74.31, 1158.44)** | **0.0259** | **645.94 (61.83,1230.06)** | **0.0302** |
| West | **1448.87 (929.42, 1968.31)** | **<0.0001** | **1486.94 (905.12,2068.77)** | **<.0001** |
| Teaching status of hospital |  |  |  |  |
| Metropolitan non -teaching or non-metro | Ref |  | Ref |  |
| Metropolitan teaching | 67.44 (-244.26, 379.13) | 0.6712 | -50.62 (-424.18,322.95) | 0.7903 |
| Comorbidities |  |  |  |  |
| CHD (ref: no) | -8.21 (-109.95, 93.53) | 0.8741 | 51.58 (-22.54,125.69) | 0.1723 |
| Hyperlipidemia (ref: no) | -83.06 (-172.67, 6.56) | 0.0692 | 73.38 (3.73,143.02) | 0.0018 |
| Renal failure (ref: no) | **-306.21 (-395.11, -217.32)** | **<0.0001** | **-297.52 (-395.70,-199.33)** | **<0.0001** |
| Heart failure (ref: no) | **-185.16 (-263.60, -106.71)** | **<0.0001** | **-151.42 (-207.76,-95.08)** | **<0.0001** |
| Gout (ref: no) | **-243.65 (-385.48, -101.81)** | **0.0008** | **-143.23 (-274.13,-12.33)** | **0.0320** |
| Diabetes (ref: no) | 49.99 (-45.49, 145.47) | 0.3044 | **94.17 (13.06,175.27)** | **0.0229** |
| Hypertension (ref: no) | 45.98 (-41.18, 133.15) | 0.3007 | **73.38 (3.73,143.02)** | **0.0389** |
| Osteoarthritis (ref: no) | **-244.30 (-365.26, -123.34)** | **0.0001** | **-228.81 (-348.88,-108.74)** | **0.0002** |
| IQR, Interquartile range; SE, standard error | | | | |

Appendix 4. Characteristics of patients with COPD ED visits with and without hospitalization

|  | **2012 NEDS (all)** | **2012 NEDS, Not admitted** | **2012 NEDS who were admitted** | **p-value*** |
| --- | --- | --- | --- | --- |
| **ED visits** | **1,100,378** | **559,996 (50.89)** | **540,382 (49.11)** |  |
|  |  |  |  |  |
| Age in years, Mean (SE) | 65.49 (0.13) | 62.75 (0.14) | 68.33 (0.12) | **<0.0001** |
| Sex |  |  |  |  |
| Female | 608,895 (55.33) | 307,704 (54.95) | 301,191 (55.74) | **<0.0001** |
| Patient location (residence) |  |  |  | **<0.0001** |
| Micropolitan/not metro | 297,501 (27.13) | 183,971 (32.96) | 113,530 (21.09) |  |
| Metropolitan (large or small) | 798,895 (72.87) | 374,129 (67.04) | 424,765 (78.91) |  |
| Median house hold income |  |  |  | **<0.0001** |
| 1st quartile (< $38,999) | 428,687 (39.81) | 238,673 (43.61) | 190,014 (35.88) |  |
| 2nd quartile ($39,000 to $47,999) | 295,576 (27.45) | 156,405 (28.58) | 139,170 (26.28) |  |
| 3rd quartile ($48,000 to $62999) | 116,337 (20.34) | 102,741 (18.77) | 116,337 (21.97) |  |
| 4th quartile ($63,000 or more) | 133,571 (12.40) | 49,501 (9.04) | 84,070 (15.87) |  |
| Primary payer |  |  |  | **<0.0001** |
| Medicare | 692,608 (63.02) | 312,497 (55.90) | 380,111 (70.39) |  |
| Medicaid | 163,123 (14.84) | 98,092 (17.55) | 65,031 (12.04) |  |
| Private insurance | 128,603 (11.70) | 70,826(12.67) | 57,777 (10.70) |  |
| Self-pay | 78,506 (7.14) | 57,566 (10.30) | 20,940 (3.88) |  |
| No charge | 6,367 (0.58) | 3,443 (0.62) | 2,924 (0.54) |  |
| Other | 29,812 (2.71) | 16,615 (2.97) | 13,197 (2.44) |  |
| Hospital Region |  |  |  | **<0.0001** |
| Northeast | 181,739 (16.52) | 73,658 (13.15) | 108,081 (20.00) |  |
| Midwest | 268,176 (24.37) | 152,875 (27.29) | 115,301 (21.34) |  |
| South | 498,801 (45.33) | 251,656 (44.94) | 247,145 (45.74) |  |
| West | 151,661 (13.78) | 81,807 (14.61) | 69,855 (12.93) |  |
| Teaching status of hospital |  |  |  | **<0.0001** |
| Metropolitan non -teaching or non-metro | 742,700 (67.49) | 398,918 (71.24) | 343,781 (63.62) |  |
| Metropolitan teaching | 357,679 (32.51) | 161,078 (28.76) | 196,601 (36.38) |  |
| Comorbidities |  |  |  |  |
| CHD | 234,482 (21.31) | 63,815 (11.40) | 170,667 (31.58) | **<0.0001** |
| Hyperlipidemia | 260,382 (23.66) | 65,577 (11.71) | 194,805 (36.05) | **<0.0001** |
| Renal failure | 86,323 (7.84) | 13,804 (2.47) | 72,519 (13.42) | **<0.0001** |
| CHF | 189,641 (17.23) | 49,878 (8.91) | 139,764 (25.86) | **<0.0001** |
| Hypertension | 561,574 (51.03) | 203,196 (36.29) | 358,378 (66.32) | **<0.0001** |
| Diabetes | 248,437 (22.58) | 89,617 (16.00) | 158,820 (29.39) | **<0.0001** |
| Gout | 16,026 (1.46) | 3,006 (0.54) | 13,020 (2.41) | **<0.0001** |
| Osteoarthritis | 54,405 (4.94) | 8,113 (1.45) | 46,292 (8.57) | **<0.0001** |
| CHD, coronary heart disease; CHF, Congestive Heart failure; COPD, chronic obstructive pulmonary disease; *p-value **comparing not admitted vs. admitted** | | | | |

Appendix 5. Outcomes of patients after hospital admission with COPD as primary or primary/secondary diagnosis

|  | **2009 NEDS** | **2010 NEDS** | **2012 NEDS** |
| --- | --- | --- | --- |
| **Duration of hospital stay, in days,** Mean (SE) |  |  |  |
| COPD as primary for those with ED visits | 4.55 (0.03) | 4.40 (0.03) | 4.28 (0.03) |
| COPD primary or secondary diagnosis for ED visit | 5.57 (0.04) | 5.38 (0.04) | 5.22 (0.04) |
| **Discharge disposition after Hospitalization for COPD as primary diagnosis,** n(%) |  |  |  |
| Discharged home | 365,167 (65.61) | 356,140 (64.83) | 353,237 (65.39) |
| Skilled nursing facility, intermediate Care facility, and another type of facility | 79,561 (14.30)) | 77,728 (14.15) | 72,142 (13.35) |
| Transferred to short term hospital | 8,184 (1.47) | 7,424 (1.35) | 6,575 (1.22) |
| Home health care | 87,035 (15.64) | 91,946 (16.74) | 92,649 (17.15) |
| Against medical advice | 8,393 (1.51) | 8,687 (1.58) | 9,458 (1.75) |
| Died | 7,949 (1.43) | 7,104 (1.29) | 6,046 (1.12) |
|  |  |  |  |
| **Discharge disposition after Hospitalization for COPD as primary or secondary diagnosis*,** n(%) |  |  |  |
| Discharged home | 1,525,113 (51.56) | 1,516,033 (50.82) | 1,511,434 (51.51) |
| Skilled nursing facility, intermediate Care facility, and another type of facility | 718,428 (24.29) | 722,964 (24.23) | 687,594 (23.43) |
| Transferred to short term hospital | 87,804 (2.97) | 85,646 (2.87) | 79,590 (2.71) |
| Home health care | 466,520 (15.77) | 501,794 (16.82) | 510,414 (17.39) |
| Against medical advice | 37,773 (1.28) | 39,605 (1.33) | 43,160 (1.47) |
| Died | 120,373 (4.07) | 114,468 (3.84) | 1,368 (0.05) |
| SE, standard error | | | |

Appendix 6. Predictors of Discharge to nursing home/skilled nursing facility among patients who were admitted to the hospital with COPD as the primary diagnosis after presenting to ED, using logistic regression

|  | Univariate | | Multivariable-adjusted | |
| --- | --- | --- | --- | --- |
|  | OR (95% CI) | P-value | OR (95% CI) | P-value |
| Age |  |  |  |  |
| <50 | Ref |  | Ref |  |
| 50- <65 | **1.95 (1.71, 2.22)** | **<0.0001** | **1.74 (1.51, 1.99)** | **<0.0001** |
| 65- <80 | **4.19 (3.65, 4.82)** | **<0.0001** | **2.88 (2.47, 3.36)** | **<0.0001** |
| ≥80 | **9.66 (8.37,11.14)** | **<0.0001** | **6.32 (5.38, 7.42)** | **<0.0001** |
| Gender |  |  |  |  |
| Female | Ref |  | Ref |  |
| Male | **0.83 (0.80, 0.86)** | **<0.0001** | **0.87 (0.83, 0.90)** | **<0.0001** |
| Median house hold income |  |  |  |  |
| 1st quartile (<$38,999) | Ref |  | Ref |  |
| 2nd quartile ($39,000 to $47,999) | 1.04 (0.96, 1.12) | 0.3392 | 0.96 (0.89, 1.04) | 0.3116 |
| 3rd quartile ($48,000 to $62999) | **1.18 (1.07, 1.30)** | **0.0009** | 1.03 (0.94, 1.13) | 0.5183 |
| 4th quartile ($63,000 or more) | **1.45 (1.33, 1.57)** | **<0.0001** | 1.06 (0.96, 1.16) | 0.2717 |
| Primary payer |  |  |  |  |
| Medicare | Ref |  | Ref |  |
| Medicaid | **0.36 (0.33, 0.40)** | **<0.0001** | **0.73 (0.66, 0.81)** | **<0.0001** |
| Private insurance | **0.34 (0.31, 0.37)** | **<0.0001** | **0.54 (0.49, 0.60)** | **<0.0001** |
| Self-pay/No charge | **0.11 (0.09, 0.14)** | **<0.0001** | **0.28 (0.22,0.34)** | **<0.0001** |
| Other | **0.48 (0.39, 0.60)** | **<0.0001** | 0.84 (0.67, 1.06) | 0.1434 |
| Patient location (residence) |  |  |  |  |
| Micropolitan/not metro | Ref |  | Ref |  |
| Metropolitan (large or small) | 1.00 (0.93, 1.08) | 0.9504 | 0.93 (0.85, 1.02) | 0.1089 |
| Hospital Region |  |  |  |  |
| Northeast | Ref |  | Ref |  |
| Midwest | **0.88 (0.79, 0.99)** | **0.0271** | 1.11 (0.98, 1.26) | 0.0980 |
| South | **0.69 (0.62, 0.76)** | **<0.0001** | **0.82 (0.72, 0.92)** | **0.0007** |
| West | **0.75 (0.66, 0.85)** | **<0.0001** | **0.85 (0.74, 0.98)** | **0.0206** |
| Teaching status of hospital |  |  |  |  |
| Metropolitan non -teaching or non-metro | Ref |  | Ref |  |
| Metropolitan teaching | **0.89 (0.81, 0.98)** | **0.0210** | **0.86 (0.78, 0.95)** | **0.0028** |
| Comorbidities |  |  |  |  |
| CHD (ref: no) | **1.21 (1.16, 1.26)** | **<0.0001** | 0.97 (0.93, 1.02) | 0.2443 |
| Hyperlipidemia (ref: no) | **0.85 (0.82, 0.89)** | **<0.0001** | **0.77 (0.74, 0.80)** | **<0.0001** |
| Renal failure (ref: no) | **1.54 (1.47, 1.61)** | **<0.0001** | 1.05 (1.00, 1.10) | 0.0604 |
| CHF (ref: no) | **1.81 (1.74, 1.87)** | **<0.0001** | **1.30 (1.25, 1.36)** | **<0.0001** |
| Gout (ref: no) | 1.04 (0.92, 1.17) | 0.5187 | **0.84 (0.74, 0.95)** | **0.0051** |
| Diabetes (ref: no) | **1.04 (1.00, 1.08)** | **0.0374** | **1.06 (1.01, 1.10)** | **0.0073** |
| Hypertension (ref: no) | **1.09 (1.05, 1.13)** | **<0.0001** | **0.95 (0.91, 0.99)** | **0.0121** |
| Osteoarthritis (ref: no) | **1.22 (1.15, 1.29)** | **<0.0001** | **1.06 (1.00, 1.13)** | **0.0458** |
| Length of stay, in days | **1.16 (1.15, 1.17)** | **<0.0001** | **1.15 (1.14, 1.16)** | **<0.0001** |
| CHD, coronary heart disease; CHF, Congestive Heart failure; COPD, chronic obstructive pulmonary disease; **Significant odds ratios are in bold** | | | | |

Appendix 7. Predictors of Log of duration of hospital stay among patients with COPD who were admitted to the hospital with COPD as the primary diagnosis after presenting to ED, using linear regression

|  | Univariate |  | Multivariable-adjusted |  |
| --- | --- | --- | --- | --- |
|  | B-estimate (95% CI) | P-value | B-estimate (95% CI) | P-value |
| Age |  |  |  |  |
| <50 | Ref |  | Ref |  |
| 50- <65 | **0.11 (0.09, 0.12)** | **<0.0001** | **0.10 (0.08, 0.11)** | **<0.0001** |
| 65- <80 | **0.20 (0.18, 0.22)** | **<0.0001** | **0.16 (0.14, 0.17)** | **<0.0001** |
| ≥80 | **0.25 (0.23, 0.27)** | **<0.0001** | **0.18 (0.16, 0.20)** | **<0.0001** |
| Gender |  |  |  |  |
| Female (ref) | Ref |  | Ref |  |
| Male | **-0.07 (-0.07,-0.06)** | **<0.0001** | **-0.07 (-0.08, -0.06)** | **<0.0001** |
| Median house hold income |  |  |  |  |
| 1st quartile (< $38,999) | Ref |  | Ref |  |
| 2nd quartile ($39,000 to $47,999) | -0.00 (-0.02, 0.01) | 0.6645 | -0.01 (-0.03, 0.01) | 0.3031 |
| 3rd quartile ($48,000 to $62999) | 0.01 (-0.01, 0.03) | 0.5211 | -0.01 (-0.03, 0.01) | 0.1804 |
| 4th quartile ($63,000 or more) | **0.06 (0.03, 0.09)** | **0.0001** | 0.01 (-0.01, 0.04) | 0.3462 |
| Primary payer |  |  |  |  |
| Medicare (ref) | Ref |  | Ref |  |
| Medicaid | **-0.09 (-0.11, -0.08)** | **<0.0001** | **-0.02 (-0.03, 0.00)** | **0.0163** |
| Private insurance | **-0.09 (-0.11, -0.08)** | **<0.0001** | **-0.04 (-0.05, -0.02)** | **<0.0001** |
| Self-pay/No charge | **-0.23 (-0.25, -0.21)** | **<0.0001** | **-0.14 (-0.16, -0.12)** | **<0.0001** |
| Other | **-0.12 (-0.15, -0.08)** | **<0.0001** | **-0.05 (-0.07, -0.02)** | **0.0023** |
| Patient location (residence) |  |  |  |  |
| Micropolitan/not metro | Ref |  | Ref |  |
| Metro (large or small) | **0.04 (0.02, 0.06)** | **0.0001** | **0.04 (0.01, 0.06)** | **0.0025** |
| Hospital Region |  |  |  |  |
| Northeast | Ref |  | Ref |  |
| Midwest | **-0.13 (-0.16, -0.09)** | **<0.0001** | **-0.11 (-0.15, -0.07)** | **<0.0001** |
| South | **-0.07 (-0.11, -0.04)** | **0.0001** | **-0.05 (-0.08, -0.01)** | **0.0067** |
| West | **-0.13 (-0.17, -0.10)** | **<0.0001** | **-0.13 (-0.17, -0.09)** | **<0.0001** |
| Teaching status of hospital |  |  |  |  |
| Metropolitan non -teaching or non-metro | Ref |  | Ref |  |
| Metropolitan teaching | 0.01 (-0.02, 0.03) | 0.5677 | -0.01 (-0.04, 0.02) | 0.4531 |
| Comorbidities |  |  |  |  |
| CHD (ref: no) | **0.04 (0.03, 0.04)** | **<0.0001** | 0.00 (-0.01, 0.00) | 0.4987 |
| Hyperlipidemia (ref: no) | **-0.01 (-0.02, -0.00)** | **0.0031** | **-0.04 (-0.05, -0.03)** | **<0.0001** |
| Renal failure (ref: no) | **0.10 (0.09, 0.11)** | **<0.0001** | **0.05 (0.04, 0.06)** | **<0.0001** |
| Heart failure (ref: no) | **0.14 (0.13, 0.15)** | **<0.0001** | **0.11 (0.10, 0.12)** | **<0.0001** |
| Hypertension (ref: no) | **0.03 (0.02, 0.03)** | **<0.0001** | -0.01 (-0.01,0.00) | 0.1335 |
| Diabetes (ref: no) | **0.03 (0.03, 0.04)** | **<0.0001** | **0.02 (0.01, 0.02)** | **0.0001** |
| Gout (ref: no) | 0.02 (-0.01, 0.04) | 0.1421 | **-0.03 (-0.05, -0.01)** | **0.0139** |
| Osteoarthritis (ref: no) | 0.03 (0.01, 0.04) | 0.0006 | 0.01 (-0.01, 0.02) | 0.2864 |
| CHD, coronary heart disease; CHF, Congestive Heart failure; COPD, chronic obstructive pulmonary disease; **Significant beta coefficients are in bold**. | | | | |

Appendix 8. Predictors of Duration of stay (length of stay >2; reference, length of stay ≤2) among patients who were admitted to the hospital with COPD as the primary diagnosis after presenting to ED, using logistic regression

|  | Univariate |  | Multivariable-adjusted |  |
| --- | --- | --- | --- | --- |
|  | OR (95% CI) | P-value | OR (95% CI) | P-value |
| Age |  |  |  |  |
| <50 | Ref |  | Ref |  |
| 50- <65 | **1.43 (1.36, 1.51)** | **<0.0001** | **1.38 (1.30, 1.46)** | **<0.0001** |
| 65- <80 | **1.94 (1.83, 2.06)** | **<0.0001** | **1.65 (1.55, 1.77)** | **<0.0001** |
| ≥80 | **2.42 (2.26, 2.59)** | **<0.0001** | **1.92 (1.78, 2.07)** | **<0.0001** |
| Gender |  |  |  |  |
| Female | Ref |  | Ref |  |
| Male | **0.78 (0.76, 0.80)** | **<0.0001** | **0.77 (0.75, 0.80)** | **<0.0001** |
| Median house hold income |  |  |  |  |
| 1st quartile (< $38,999) | Ref |  | Ref |  |
| 2nd quartile ($39,000 to $47,999) | 0.95 (0.89, 1.02) | 0.1499 | 0.93 (0.88, 1.00) | 0.0436 |
| 3rd quartile ($48,000 to $62999) | 1.01 (0.94, 1.08) | 0.8697 | 0.95 (0.88, 1.02) | 0.1385 |
| 4th quartile ($63,000 or more) | 1.15 (1.04, 1.27) | 0.0057 | 0.98 (0.89, 1.08) | 0.7291 |
| Primary payer |  |  |  |  |
| Medicare | Ref |  | Ref |  |
| Medicaid | **0.70 (0.67, 0.74)** | **<0.0001** | **0.90 (0.85, 0.95)** | **0.0004** |
| Private insurance | **0.71 (0.67, 0.75)** | **<0.0001** | **0.87 (0.82, 0.92)** | **<0.0001** |
| Self-pay/No charge | **0.45 (0.42, 0.48)** | **<0.0001** | **0.62 (0.57, 0.66)** | **<0.0001** |
| Other | **0.68 (0.60, 0.77)** | **<0.0001** | **0.87 (0.77, 0.97)** | **0.0115** |
| Patient location (residence) |  |  |  |  |
| Micropolitan/ not metro | Ref |  | Ref |  |
| Metro (large or small) | 1.08 (1.00, 1.16) | 0.0557 | **1.10 (1.01, 1.19)** | **0.0255** |
| Hospital Region |  |  |  |  |
| Northeast | Ref |  | Ref |  |
| Midwest | **0.69 (0.61, 0.79)** | **<0.0001** | **0.72 (0.63, 0.82)** | **<0.0001** |
| South | **0.80 (0.71, 0.91)** | **0.0003** | **0.84 (0.75, 0.95)** | **0.0058** |
| West | **0.68 (0.60, 0.77)** | **<0.0001** | **0.67 (0.58, 0.76)** | **<0.0001** |
| Teaching status of hospital |  |  |  |  |
| Metropolitan non -teaching or non-metro | Ref |  | Ref |  |
| Metropolitan teaching | 0.94 (0.87, 1.02) | 0.1612 | **0.90 (0.83, 0.99)** | **0.0245** |
| Comorbidities |  |  |  |  |
| CHD (ref: no) | 1.14 (1.11, 1.18) | **<0.0001** | 0.99 (0.96, 1.02) | 0.6736 |
| Hyperlipidemia (ref: no) | 1.01 (0.98, 1.05) | 0.4364 | **0.91 (0.88, 0.94)** | **<0.0001** |
| Renal failure (ref: no) | **1.40 (1.34, 1.46)** | **<0.0001** | **1.16 (1.11, 1.22)** | **<0.0001** |
| Congestive Heart failure (ref: no) | **1.54 (1.49, 1.60)** | **<0.0001** | **1.39 (1.34, 1.44)** | **<0.0001** |
| Hypertension (ref: no) | **1.15 (1.12, 1.18)** | **<0.0001** | 1.02 (0.99, 1.06) | 0.1768 |
| Diabetes (ref: no) | **1.17 (1.13, 1.21)** | **<0.0001** | **1.10 (1.06, 1.13)** | **<0.0001** |
| Gout (ref: no) | 1.06 (0.97, 1.15) | 0.1914 | **0.91 (0.84, 0.99)** | **0.0312** |
| OA (ref: no) | **1.20 (1.14, 1.28)** | **<0.0001** | **1.10 (1.04, 1.17)** | **0.0007** |
| CHD, coronary heart disease; CHF, Congestive Heart failure; COPD, chronic obstructive pulmonary disease; **Significant odds ratios are in bold** | | | | |
|  |  |  |  |  |

Appendix 9. Predictors of **log of Total hospital charges** (ED plus inpatient) among patients with COPD who were admitted to the hospital using linear regression

|  | Univariate |  | Multivariable-adjusted |  |
| --- | --- | --- | --- | --- |
|  | B-estimate (95% CI) | P-value | B-estimate (95% CI) | P-value |
| Age |  |  |  |  |
| <50 | Ref |  | Ref |  |
| 50- <65 | **0.14 (0.11,0.16)** | **<0.0001** | **0.11 (0.08,0.13)** | **<0.0001** |
| 65- <80 | **0.23 (0.20,0.26)** | **<0.0001** | **0.14 (0.11,0.17)** | **<0.0001** |
| ≥80 | **0.27 (0.22,0.31)** | **<0.0001** | **0.13 (0.09,0.17)** | **<0.0001** |
| Gender |  |  |  |  |
| Female | Ref |  | Ref |  |
| Male | **-0.03 (-0.04,-0.01)** | **0.0001** | **-0.04 (-0.05,-0.02)** | **<0.0001** |
| Median house hold income |  |  |  |  |
| 1st quartile (< $38,999) | Ref |  | Ref |  |
| 2nd quartile ($39,000 to $47,999) | -0.01 (-0.07,0.06) | 0.7924 | -0.03 (-0.09,0.03) | 0.3845 |
| 3rd quartile ($48,000 to $62999) | **0.12 (0.05,0.20)** | **0.0015** | 0.05 (-0.02,0.12) | 0.1718 |
| 4th quartile ($63,000 or more) | **0.18 (0.07,0.30)** | **0.0020** | 0.11 (-0.01,0.23) | 0.0745 |
| Primary payer |  |  |  |  |
| Medicare | Ref |  | Ref |  |
| Medicaid | **-0.09 (-0.12,-0.05)** | **<0.0001** | **-0.05 (-0.08,-0.02)** | **0.0032** |
| Private insurance | **-0.15 (-0.18,-0.11)** | **<0.0001** | **-0.09 (-0.12,-0.05)** | **0.0000** |
| Self-pay/No charge | **-0.22 (-0.26,-0.18)** | **<0.0001** | **-0.12 (-0.16,-0.08)** | **0.0000** |
| Other | **-0.08 (-0.15,-0.01)** | **0.0297** | **-0.08 (-0.13,-0.02)** | **0.0105** |
| Patient location (residence) |  |  |  |  |
| Micropolitan/not metro | Ref |  | Ref |  |
| Metro (large or small) | **0.30 (0.22,0.38)** | **<0.0001** | **0.26 (0.18,0.34)** | **<0.0001** |
| Hospital Region |  |  |  |  |
| Northeast | Ref |  | Ref |  |
| Midwest | -0.07 (-0.22,0.07) | 0.3079 | -0.01 (-0.15,0.13) | 0.9189 |
| South | 0.09 (-0.06,0.23) | 0.2550 | **0.16 (0.01,0.31)** | **0.0365** |
| West | **0.57 (0.42,0.72)** | **<0.0001** | **0.61 (0.46,0.76)** | **<0.0001** |
| Teaching status of hospital |  |  |  |  |
| Metropolitan non -teaching or non-metro | Ref |  | Ref |  |
| Metropolitan teaching | 0.02 (-0.09,0.12) | 0.7721 | -0.00 (-0.11,0.11) | 0.9864 |
| Comorbidities |  |  |  |  |
| CHD (ref: no) | **0.08 (0.07,0.10)** | **<0.0001** | **0.04 (0.03,0.05)** | **<0.0001** |
| Hyperlipidemia (ref: no) | -0.01 (-0.03,0.01) | 0.1795 | **-0.04 (-0.06,-0.02)** | **<0.0001** |
| Renal failure (ref: no) | **0.16 (0.14,0.18)** | **<0.0001** | **0.07 (0.05,0.09)** | **<0.0001** |
| Heart failure (ref: no) | **0.21 (0.19,0.23)** | **<0.0001** | **0.16 (0.15,0.18)** | **<0.0001** |
| Hypertension (ref: no) | **0.06 (0.05,0.08)** | **<0.0001** | **0.02 (0.00,0.04)** | **0.0128** |
| Diabetes (ref: no) | **0.07 (0.06,0.09)** | **<0.0001** | **0.05 (0.03,0.06)** | **<0.0001** |
| Gout (ref: no) | 0.00 (-0.03,0.04) | 0.8672 | **-0.06 (-0.10,-0.03)** | **0.0004** |
| OA (ref: no) | -0.03 (-0.06,0.00) | 0.0619 | **-0.03 (-0.06,-0.00)** | **0.0233** |
| CHD, coronary heart disease; CHF, Congestive Heart failure; COPD, chronic obstructive pulmonary disease; **Significant beta coefficients are in bold** | | | | |
